# Antibiofilm Activity, Compound Characterization, and Acute Toxicity of Extract from a Novel Bacterial Species of *Paenibacillus*

**DOI:** 10.1155/2014/649420

**Published:** 2014-03-24

**Authors:** Saad Musbah Alasil, Rahmat Omar, Salmah Ismail, Mohd Yasim Yusof

**Affiliations:** ^1^Department of Microbiology, Faculty of Medicine, MAHSA University, 59100 Kuala Lumpur, Malaysia; ^2^Pantai Hospital Cheras, 56100 Kuala Lumpur, Malaysia; ^3^Institute of Biological Science, Faculty of Science, University of Malaya, 50603 Kuala Lumpur, Malaysia; ^4^Department of Medical Microbiology, Faculty of Medicine, University of Malaya, 50603 Kuala Lumpur, Malaysia

## Abstract

The effectiveness of many antimicrobial agents is currently decreasing; therefore, it is important to search for alternative therapeutics. Our study was carried out to assess the *in vitro* antibiofilm activity using microtiter plate assay, to characterize the bioactive compounds using Ultra Performance Liquid Chromatography-Diode Array Detection and Liquid Chromatography-Mass Spectrometry and to test the oral acute toxicity on Sprague Dawley rats of extract derived from a novel bacterial species of *Paenibacillus* strain 139SI. Our results indicate that the crude extract and its three identified compounds exhibit strong antibiofilm activity against a broad range of clinically important pathogens. Three potential compounds were identified including an amino acid antibiotic C_8_H_20_N_3_O_4_P (MW 253.237), phospholipase A2 inhibitor C_21_H_36_O_5_ (MW 368.512), and an antibacterial agent C_14_H_11_N_3_O_2_ (MW 253.260). The acute toxicity test indicates that the mortality rate among all rats was low and that the biochemical parameters, hematological profile, and histopathology examination of liver and kidneys showed no significant differences between experimental groups (*P* > 0.05). Overall, our findings suggest that the extract and its purified compounds derived from novel *Paenibacillus* sp. are nontoxic exhibiting strong antibiofilm activity against Gram-positive and Gram-negative pathogens that can be useful towards new therapeutic management of biofilm-associated infections.

## 1. Introduction 

The effectiveness of many antimicrobial agents is currently decreasing due to the prevalence of multidrug-resistant pathogens [1]. The emerging of these pathogens remains a serious challenge to medicine and healthcare [2]. One of the mechanisms for such resistance is the formation of biofilms which are layers of microbial cells attached to a surface and embedded in a matrix of exopolysaccharide [3]. Therefore, it is important to search for alternative therapeutics to control biofilm-associated infections. Although several plant-based compounds are receiving attention for their therapeutic properties [4], only few are reported to exhibit antibiofilm activity [5]. Natural ecosystems are rich sources of microbes that produce a wide range of compounds that exhibit diverse and versatile biological effects [6, 7]. Many marine and soil microorganisms were recently documented for their effective antibiofilm property against pathogens [8–10]. The genus* Paenibacillus* represents one of the important soil bacteria that comprise strains of medical, industrial, and agricultural importance [11]. Interest in* Paenibacillus *species as a source of new antimicrobials has been increasing and the probability of finding novel antibiofilm compounds from these bacterial strains is promising [2]. It is worth mentioning that the administration of antimicrobial agents and biocide compounds in the local sites of some infection has been a useful approach to combat microbial biofilms [12]. However, prolonged persistence of these compounds in the environment could induce toxicity towards nontarget organisms and resistance among microorganisms within biofilms [13]. Moreover, some of these compounds may exhibit toxic effects even at therapeutic doses which makes it necessary to test their toxicity in experimental animals [14]. This aspect has led to the development of more environment friendly compounds to combat with the issue. Acute toxicity is the toxicity produced by a compound when it is administered in one or more doses during a period of 24 hours [15]. These studies are usually necessary for any compound intended for human use and the information obtained from them is useful in identifying the organs of toxicity and choosing the safe doses [15]. The objective of acute studies can usually be achieved in rodents using small groups of experimental animals [15]. Therefore, our study was carried out to assess the* in vitro *antibiofilm activity, characterize the bioactive compounds, and test the acute toxicity on Sprague Dawley rats of an extract derived from novel bacterial species of* Paenibacillus *strain 139SI.

## 2. Materials and Methods

### 2.1. Bacterial Isolates

The clinical bacterial isolates were collected from patients undergoing tonsillectomy for chronic and recurrent tonsillitis at University Malaya Medical Centre (UMMC) upon approval by the medical ethics committee (PPUM/UPP/300/02/02 reference number 744.11). Reference bacterial strains used were* Staphylococcus aureus* (ATCC 25923),* Pseudomonas aeruginosa* (ATCC 27853), and* Escherichia coli* (ATCC 25922) [16]. A bacterial strain 139SI originally isolated from a local agricultural soil was identified as* Paenibacillus* via 16S rRNA gene sequencing and deposited at the American Type Culture Collection (ATCC) with a cataloguing number (ATCC–BAA-2268) [17].

### 2.2. Experimental Animals

A total of 36 adult male and female Sprague Dawley (SD) rats were obtained from the Animal Care Unit Center (ACUC) at the Faculty of Medicine, University of Malaya. Animals were weighing 150–200 gm and were kept in wire-bottomed cages at 25°C temperature, 50% humidity, and a 12-hour light-dark cycle for at least 3 days before the experiment to allow for their acclimatization to the conditions of experiments. Animals were maintained at standard housing conditions and free access to standard diet and water* ad libitum* during the experiment. The experimental protocol was approved by the Animal Ethics Committee (PM/27/07/2010/MAA (R)) and all animals received humane care according to the guide for the care and use of laboratory animals [18] and the guide for the control of experiments on animals (CPCSEA) [19].

### 2.3. Preparation of* Paenibacillus* sp. Cell-Free Supernatant

A single colony from the culture of* Paenibacillus *species strain 139SI was transferred into sterile brain heart infusion (BHI) broth (BD Difco) followed by incubation at 37°C. We have prepared the growth curve of* Paenibacillus* 139SI supernatant (extract) in three different incubation periods after 24, 48, and 72 hours. However, only the 72-hour extract showed the highest activity compared to the 24 and 48 hours. This was due to the longer incubation period that allows the maximum secretion of bioactive metabolites by the* Paenibacillus* 139SI colonies into the culture media. Therefore, only the 72-hour incubation extract was used in our study. The* Paenibacillus *extract was then transferred aseptically into 50 mL conical bottom centrifuge tube (Jet Biofil) followed by centrifugation at 8000 rpm in 4°C for 20 min to separate the cell from the supernatant. The obtained supernatant was then subjected to sterile filtration to remove all unwanted particles using syringe filter with a pore size of 0.22 *μ*m (Minisart Sartorius) [20]. The obtained cell-free supernatant was then freeze-dried and dissolved in ultra-pure water (MilliQ, Millipore) and stored at −20°C as a stock to be used for all experiments. For each 1 mg freeze-dried supernatant powder, the amount of ultra-pure water used to resuspend the powder was 1 mL.

### 2.4. *In Vitro* Antibiofilm Activity

To assess the antibiofilm activity of* Paenibacillus* sp. strain 139SI extract and its purified compounds against clinically important pathogens, microtiter plate (MTP) assay was carried out using 96-well flat bottom polystyrene titer plates as described previously [21, 22]. Each well was filled with 100 *μ*L sterile BHI broth and 50 *μ*L overnight culture for each clinical pathogenic isolate followed by adding 150 *μ*L* Paenibacillus* sp. crude extract and 150 *μ*L of its purified compounds separately with concentrations of 1000, 1500, 2000, 2500, 3000, 3500, 4000, and 4500 *μ*g/mL before incubation at 37°C for 24 hours. After incubation, plates were gently washed three times with phosphate-buffered saline and the planktonic cells were discarded while the weakly adherent cells were removed through two rounds of thorough washing with deionized water and allowed to air-dry before being stained. The adherent biofilm was stained by 200 *μ*L of 0.4% crystal violet solution* (w/v)* for 10 min. The optical density (OD) of the biofilm was measured at 570 nm (OD_570_) with a microtiter absorbance reader (iMark, Bio-Rad) [23]. To compensate for possible differences in growth rates under the different incubation conditions and/or for strains with different characteristics, the adherence index was adjusted as an estimate of the density of the biofilm which would be generated by a culture with an OD_600_ of 0.5 [24]. Calculation of the adherence index was done according to the following formula: adherence index = mean density of biofilm (OD_570_) × 0.5/mean growth (OD_600_).

### 2.5. Biofilm Inhibitory Concentration

In order to determine the lowest concentration of strain 139SI extract that can cause visible inhibition in the biofilm formation, the biofilm inhibitory concentration (BIC) test was carried out using 6-well flat bottom polystyrene titer plates as described previously with few modifications [25]. A piece of glass cover slip (1 × 1 cm) was placed inside each well to allow the growth of bacterial isolates on the surface and to visualize the inhibitory effect of 139SI extract on the biofilm formation. Each well was filled with 300 *μ*L sterile BHI broth followed by inoculation with 150 *μ*L of overnight culture for each clinical pathogenic isolate then addition of 150 *μ*L* Paenibacillus* sp. extract and 150 *μ*L of its purified compounds separately with concentrations ranging from 1000 to 4500 *μ*g/mL before incubation at 37°C for 24 hours. After incubation, biofilm inhibition was determined spectrophotometrically using a microtiter absorbance reader (iMark, Bio-Rad) and visualized microscopically using an upright light microscope (Eclipse LV150L, Nikon).

### 2.6. Characterization of Bioactive Compounds

The* Paenibacillus *sp. cell-free supernatant was subjected to High Performance Liquid Chromatography (HPLC). Briefly, the extract solution was filtered using an SRP-4 membrane 0.45 *μ*m and injected into the HPLC column (Agilent Zorbax XDB-C18, 4.6 × 250 mm, 5.0 *μ*m) at a 100 *μ*L injection volume with a flow rate of 1.2 mL/min. The standard solvent system was a combination of acetonitrile and water at a pH of 3.55. Furthermore, the spectrum range was 200–500 nm with UV absorption of 200, 230, 254, and 320 nm. Data acquisition time was between 0 and 32 min yielding a total of 32 fractions (compounds). Further analysis to identify the chemical structure of each of the purified fractions was conducted using Ultra Performance Liquid Chromatography-Diode Array Detection (UPLC–DAD) and Liquid Chromatography-Mass Spectrometry (LC-MS). An Acquity UPLC system (Waters Corporation) equipped with a photo diode array detection detector was used for the analysis and quantification. The UPLC–ESI-MS peak identification was recorded using the UPLC system coupled with a LCQ DECA plus mass spectrometer equipped with an electrospray interface (Thermo-Finnigan Corporation). The quantification of UPLC–DAD was performed on a reversed-phase column Acquity UPLC BEH C-18 (2.1 × 50 mm) with 1.7 *μ*m spherical porous particles. The UPLC–ESI-MS analysis was operated in positive ESI modes and compounds were identified on the basis of their UV spectra and MS fragmentation patterns by searching the dictionary of natural products on DVD, Version 20:2 (CRC Press, Taylor & Francis Group).

### 2.7. Acute Toxicity Procedure

The virulence of our novel bacterial species of* Paenibacillus* strain 139SI was tested in experimental mice using the LD_50_ test as described previously [26]. For acute toxicity test, the selected experimental rats were randomly divided into six groups of six rats each as the following:

 group 1: normal saline (5 mL/Kg, oral) daily for 14 days (male control group),
 group 2: normal saline (5 mL/Kg, oral) daily for 14 days (female control group),
 group 3: 139SI extract (5 mL/Kg, oral) with a concentration of (2 gm/Kg) daily for 14 days (male low dose group),
 group 4: 139SI extract (5 mL/Kg, oral) with a concentration of (2 gm/Kg) daily for 14 days (female low dose group),
 group 5: 139SI extract (5 mL/Kg, oral) with a concentration of (4 gm/Kg) daily for 14 days (male high dose group),
 group 6: 139SI extract (5 mL/Kg, oral) with a concentration of (4 gm/Kg) daily for 14 days (male high dose group).

The body weight of all animals was measured daily. Mortalities, clinical signs, and time of onset were recorded. In addition, gross general observations were observed on the basis of behavioral signs such as food intake, salivation, muscular weakness, reflexes, piloerection, respiration (dyspnea), convulsion, and changes in locomotion [27]. All rats were sacrificed 24 hours after the last oral administration and overnight fasting prior to anesthesia with an intramuscular combination of Ketamine and Xylazine (1 mL of 100 mg/mL Xylazine and 9 mL of 100 mg/mL Ketamine) given at a dose of 0.1 mL/100 gm of body weight followed by necropsy. Blood samples were collected and the liver and kidneys were harvested, washed in normal saline, blotted with filter paper, and weighed. Gross examination was conducted in a blind fashion to examine the macroscopic abnormalities on the organs compared to the control. Moreover, liver and kidneys were subsequently subjected to a histopathological evaluation to examine the microscopic abnormalities on the organs compared to the control.

### 2.8. Biochemical Parameters and Hematological Profile

Upon sacrifice, blood was drawn from the jugular vein under anesthesia and samples were immediately collected and then referred to the clinical diagnostic laboratories (CDL) at University Malay Medical Centre (UMMC) for assessment of the biochemical parameters and hematological profile. For the biochemical parameters, blood was collected into yellow caped VACUETTE clot activator. Liver function tests were assessed including total protein, albumin, globulin, total bilirubin, conjugate bilirubin, alkaline phosphatase, alanine aminotransferase, aspartate aminotransferase, and g-glutamyltransferase. In addition, renal function tests were assessed including sodium, potassium, chloride, carbon dioxide, anion gap, urea, and creatinine. For the hematological profile, blood was collected into violet caped VACUETTE EDTA tubes and the complete blood count (CDC) test was assessed including hemoglobin (HGB), hematocrit (HCT), red blood cells (RBC), mean corpuscular volume (MCV), mean corpuscular hemoglobin (MCH), mean corpuscular hemoglobin concentration (MCHC), red blood cell distribution width (RDW), white blood cell (WBC), and platelet. In addition, differential blood count test was assessed including neutrophil, lymphocyte, monocyte, eosinophil, and basophil.

### 2.9. Histopathology Examination

Upon sacrifice, the thoracic cavity was opened by an excision through the peritoneum that was extended through the sternum and the rib cage was fully opened followed by the collection of liver and kidneys. The collected organs were fixed with 10% neutral buffered formalin (NBF) for 24 hours and then sliced into smaller pieces to be fixed again with NBF for another 24 hours. Histopathology examination was performed as described previously [28]. Briefly, fixed tissues were embedded in paraffin wax using an embedding center (Leica EG1160, Leica Biosystems), sectioned using a microtome (Leica RM2135, Leica Biosystems), and fixed onto glass slides using a water bath (Leica HI1210, Leica Biosystems). The paraffin sections were then stained with hematoxylin and eosin (H&E) stain mounted with diphenyl xylene (DPX) and visualized using an upright light microscope (Eclipse LV150L, Nikon).

### 2.10. Statistical Analysis

Statistical analysis was carried out using the Statistical Product and Service Solutions software (IBM SPSS statistics 21). Categorical data were compared by the *χ* ^2^ test, while unpaired differences in continuous data were compared by both the Mann-Whitney *U* test and the analysis of variance (ANOVA) test. All values were reported as standard error mean (S.E.M ±) and a probability value of *P* < 0.05 was considered to be statistically significant.

## 3. Results

### 3.1. *In Vitro *Antibiofilm Activity

The results of MTP assay for the crude extract of* Paenibacillus* sp. strain 139SI showed significant inhibition of the biofilm formation when assessed spectrophotometrically. The lowest and most effective concentration that caused the reduction in the biofilm's adherence index was 4500 *μ*g/mL. Among all the 32 purified fractions (compounds) of the crude extract, only 3 compounds showed the highest antibiofilm activity selected against Gram-negative and Gram-positive clinical isolates (Tables 1 and 2), respectively. Moreover, compound number 5 (FR5) was the most active with significant decrease in the adherence index when compared to other compounds and controls. The results of MTP assays were compatible with the BIC test in which there was an 80% inhibition in the biofilm when visualized under light microscope showing scattered bacterial cells with no extracellular matrix.

### 3.2. Characterization of Potential Compounds

The results of characterizing the compounds from* Paenibacillus *sp. extract using HPLC showed a total of 32 purified fractions in which only 3 fractions exhibited antibiofilm activity* in vitro* when assessed spectrophotometrically [29]. From these 3 fractions, a total of 3 potential compounds were identified in which the first compound was Leucine 2-(hydroxymethoxyphosphinyl)-2-methylhydrazide with a molecular weight of 253.237 and a molecular formula of C_8_H_20_N_3_O_4_P described as an amino acid antibiotic with an activity against Gram-positive and Gram-negative bacteria. The second compound was 4-Hydroxy-5-(hydroxymethyl)-3-(14-methylpentadecanoyl) tetronicacid-2(5H)-furanone with a molecular weight of 368.512 and a molecular formula of C21H36O5 described as a phospholipase A2 inhibitor. The third compound was 6-(hydroxymethyl)-1-phenazinecarboxyamide with a molecular weight of 253.260 and a molecular formula of C_14_H_11_N_3_O_2_ described as an antibacterial agent.

### 3.3. Gross General Observation of Experimental Rats

Gross general observations showed that experimental rats grew at relatively constant rates. Following the 14 days oral ingestion of* Paenibacillus* sp. extract, there was no significant difference (*P* > 0.05) in the overall growth among the groups except for high dose group where a decrease in growth was observed in the last two days of experiment. These results suggested that the 14 days acute oral ingestion of extract did not affect the weight of rats. Moreover, there was an irregular dose-dependent mortality in both sexes for which only one rat from each sex died after 72 hours ingestion of the high dose (1 out of 6 males and 1 out of 6 females). Moreover, the observed symptoms of toxicity included minor hypoactivity, loss of appetite, hyperventilation, convulsion, dizziness, and salivation; however, they were statistically insignificant when compared to the controls.

### 3.4. Biochemical Parameters and Hematological Profile of Blood

Despite minor discrepancies between sexes, the results of biochemical parameters showed no significant differences (*P* > 0.05) in the liver and kidney function tests among males and females (Tables 3 and 4), respectively. However, there were elevated levels in globulin, alkaline phosphatase, alanine aminotransferase, aspartate aminotransferase, g-glutamyltransferase, potassium, urea, and creatinine. Moreover, there were increased levels of anion gap among female rats only. Overall, our results indicate that the 139SI extract has no detectable differences on both liver and kidney functions. Moreover, the results of hematological profile (Tables 5 and 6) showed no significant differences (*P* > 0.05) in both the complete and differential blood count except for elevated levels in red blood cells (RBC), white blood cell (WBC), platelet, neutrophil, lymphocyte, and monocyte particularly among the high dose groups (4 gm/Kg).

### 3.5. Histopathology Examination of Organs

**Figure 1:**
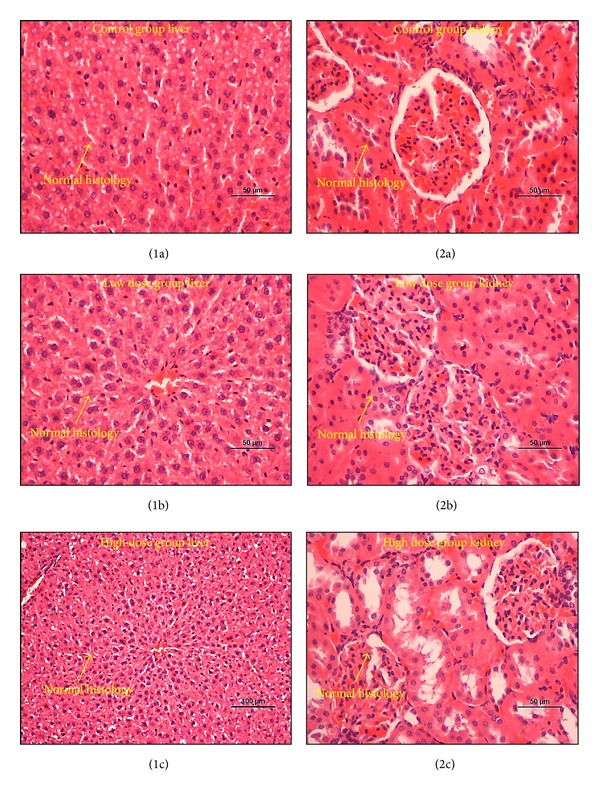
Microscopic images of representative tissue sections showing the histopathology evaluation of the organs of toxicity among SD rat groups. (1a) and (2a) are vehicle (control) groups with normal histology of liver and kidneys, respectively. (1b) and (2b) are low dose (2 gm/Kg) groups with normal histology of liver and kidneys, respectively. (1c) and (2c) are high dose (4 gm/Kg) groups with normal histology of liver and kidneys, respectively. Sections were stained with H&E stain.

Upon histological examination of liver and kidneys, the organs showed normal architecture, no changes in colour, and no morphological disturbances. Liver tissue sections showed regular cellular architecture with distinct hepatic cells, sinusoidal spaces, and a central vein. Ordinary patterns with normal parenchyma and reduced fibrous septa and lymphocyte infiltration were seen (Figure 1). Overall, the examination showed no detectable differences in the integrity of tissue among all groups and that the 139SI extract had no effects on the cellular structures and thus does not cause degeneration of cells in these particular organs.

## 4. Discussion

Bacteria that inhabit the soil are potential sources for the isolation of novel antibiofilm compounds [30]. It has been estimated that, among all the microbes isolated from soil,* Bacillus* and* Paenibacillus* species are the most frequently found members with antimicrobial and antibiofilm activities [31, 32]. Therefore, the report of a taxonomically novel species of* Paenibacillus* strain 139SI having antibiofilm activity is not surprising. Our study demonstrates the occurrence of a broad range antibiofilm activity in the crude extract and in three identified compounds of an extract from a novel* Paenibacillus *sp. strain 139SI. These identified compounds included an amino acid antibiotic, phospholipase A2 inhibitor, and antibacterial agent. To our knowledge, no literature has reported the finding of such compounds with such activity from the* Paenibacillus* species. The effect of the characterized bioactive compounds results in inhibition of the biofilm formation among the selected Gram-positive and Gram-negative isolates. This broad spectrum activity might help* Paenibacillus* sp. in the soil environment to establish itself on the surface of plant roots and critically influence the development of unique bacterial community.

It has been previously reported that some bacterial compounds such as extracellular polysaccharides (EPS) can be involved in the antibiofilm activity. For example, EPSs from the marine bacterium* Vibrio* sp. QY101 display selective or broad spectrum antibiofilm activity [33]. However, the potentiality of the compounds described in this study against a wide range of pathogenic and nonpathogenic organisms suggests that these compounds might be powerful alternatives among the previously identified compounds. Based on the findings, the first compound reported here as an amino acid antibiotic with the name Leucine 2-(hydroxymethoxyphosphinyl)-2-methylhydrazide has a phosphate group in it and, thus, it can be proposed that its electronegative property might modulate the surface of the tested organism in such a way that there is an inhibition of the cell-surface attachment. This was similar to a previous study where it was reported that the identified polysaccharide compounds might interfere with the cell-surface influencing cell-cell interactions of a wide range of bacterial isolates [13]. The second compound reported here as a phospholipase A2 inhibitor with the name 4-Hydroxy-5-(hydroxymethyl)-3-(14-methylpentadecanoyl) tetronicacid-2(5H)-furanone has a similar chemical structure to the previously identified quorum-sensing antagonist (5Z)-4-bromo-5-(bromomethylene)-3-butyl-2(5H)-furanone from the marine alga* Delisea pulchra* which was reported to inhibit the biofilm formation in* E. coli* without inhibiting its growth [34]. The third compound reported here as an antibacterial agent with the name 6-(hydroxymethyl)-1-phenazinecarboxyamide might modify the physicochemical characteristics and the architecture of the outer membrane of biofilm-forming organisms which is the phenomenon observed for some antibiotics as reported previously [35]. In the BIC test that included the use of cover slip, a gradual decrease of biofilm development was visualized with the increase of the concentration of crude extract from* Paenibacillus* sp. strain 139SI.

In recent years, many studies have focused on the acute toxicity of antimicrobial metabolites isolated from different soil microorganisms for the purpose of identifying new sources of bioactive compounds [36, 37]. Acute toxicity studies in experimental animals are useful to provide the primary data supporting single dose safety and kinetic in humans [15]. In our study, the oral acute toxicity and compound characterization of an antibiofilm extract from a novel bacterial species of* Paenibacillus* strain 139SI was assessed in Sprague Dawley (SD) rats. In the present study, a total of 36 rats were selected in which 12 rats of both sexes were treated with a low dose (2 gm/Kg) and 12 rats were treated via a gastrogavage with a high dose (4 gm/Kg) of an antibiofilm extract from the novel bacterial species of* Paenibacillus* strain 139SI at a concentration of 4500 *μ*g/mL for a duration of 14 days. In rodents, a decrease in food and water consumption is an important sign of health deterioration which generally results in the loss of bodyweight [38]. Changes in bodyweight have also been used as an indicator of the effect of drugs and chemicals [39]. Overall, the gross general observation indicated that the effect of orally administered extract was not affected by sex and that the mortality rate was low among experimental rats. These finding were similar to a previous study in which the effects of a new compound from novel soil bacterial species of* Streptomyces* were investigated on Long Evan's rats showing no adverse effects at a dose of 300 *μ*g/rat/day [27]. It is known that both liver and kidney play significant roles in various metabolic processes. However, if too many demands are made on the capacities of these organs, the function of their cells will eventually be adversely affected [40]. The liver plays an important role in xenobiotic function whereas the kidneys are the main organs involved in drugs elimination [41]. Moreover, the enzymes alanine aminotransferase (ALT) and aspartate aminotransferase (AST) are usually used as biomarkers to predict possible toxicity in the liver [42]. Therefore, any damage to the parenchymal liver cells will result in elevations in both of these enzymes [43]. In our study, the elevated levels of ALT and AST especially among high dose groups (4 gm/Kg) seemed to suggest that the* Paenibacillus* extract did affect the liver cells' mitochondria. However, this appeared to be an acute and short lasting response that did not cause significant mortalities among experimental groups. This was similar to a previous study in which the acute oral administration of an aqueous plant extract of* Artemisia afra* to mice induced the same insignificant symptoms in both sexes. Furthermore, it was noticed that the levels of anion gap increased among female rats only. Although the significance of this result is unclear, determining the concentration of anion gap may be susceptible to specific errors such as the delay in processing blood samples after collection or when particular pathological condition occurs like diarrhea that will eventually cause dehydration which will eventually lead to some alterations in the renal function. Hematopoietic system is one of the most sensitive targets for toxic compounds [44]; it was thus important to investigate the effect of our* Paenibacillus* sp. extract on the hematological profile. Our results showed that there were no significant differences (*P* > 0.05) in the haematocrits, mean cell haemoglobin concentration, platelet, and RBC and WBC counts among all experimental rats. The hemoglobin and the RBC levels were not affected suggesting that haemolytic anemia and polycythemia, that are characterized by decreases and increases in RBC count, haematocrits, and hemoglobin, respectively, were not likely to be induced by the extract. The platelet levels despite being slightly elevated were also not significant indicating that the extract also did not affect the production of platelets nor induced thrombocytopenia, the latter normally being the first evidence of drug-induced toxic effects on haematopoiesis [40]. Moreover, the levels of WBC which serve as scavengers that destroy microorganisms at infection sites [45] were also not changed suggesting that the extract was also not toxic to the immune system and did not affect leucopoiesis. Collectively, all the results suggest that the acute ingestion of the extract of novel species of* Paenibacillus* strain 139SI did not alter the haematological parameters of our SD rats. Upon visual histological examination of both liver and kidneys, the acute oral administration of the extract had no adverse effects on these organs and that it was well tolerated over the 14 days of study period. Therefore, it is being considered as the material that should be safe for use in oral formulations on preclinical and clinical studies. The bioactive compounds produced by bacteria in natural environments could be a mixture of several classes of chemical compounds that can be either amino acids, peptides, nucleosides, alkaloids, terpenoids, sterols, saponins, or polycyclic [46]. There is little information in the literature on the toxic or lethal levels of crude extract from bacteria belonging to the genus* Paenibacillus*. Our extract did not show any toxicity against experimental rats; the more likely explanation is that the toxic compounds in the crude extract were very low to induce death. However, some of the rats did die from ingesting the high dose of extract. This can be due to high concentrations of one or more of the three chemical compounds characterized by UPLC–ESI-MS. Moreover, our study raises the following concerns. Firstly, although the acute oral doses of* Paenibacillus *sp. extract did not produce any significant adverse effects in rats, further studies using higher doses of the characterized compounds may be needed. Secondly, to confirm the nontoxic nature of the extract and its derivative compounds, the effect of various factors such as type of soil, bacterial growth stage, type of growth media, and storage conditions may also need to be investigated. Thirdly, the effects of extract on the reproductive capacity of animals and on causing tumors need to be assessed.

Overall, this study provides preliminary data on the toxicity profile and potential bioactive compounds of an antibiofilm extract from a novel soil bacterial species of* Paenibacillus *that can be useful for the planning of future preclinical and clinical studies towards new therapeutic management of biofilm-associated infections.

## Figures and Tables

**Table 1:** Antibiofilm activity of potential compounds of 139SI against Gram-negative clinical isolates.

Experimental treatment	*Haemophilus influenzae *	*Haemophilus parainfluenzae *	*Klebsiella pneumoniae *	*Pseudomonas aeruginosa *	*Citrobacter *sp.	Biofilm-forming strain *P. aeruginosa *(ATCC 27853)	Nonbiofilm-forming strain *E. coli * (ATCC 25922)
	OD ± SD	OD ± SD	OD ± SD	OD ± SD	OD ± SD	OD ± SD	OD ± SD
With compound FR4	0.235 ± 0.005	0.245 ± 0.004	0.266 ± 0.004	0.194 ± 0.003	0.175 ± 0.004	0.225 ± 0.004	0.164 ± 0.004
Without compound	0.311 ± 0.002	0.459 ± 0.015	0.369 ± 0.056	0.439 ± 0.052	0.235 ± 0.015	0.539 ± 0.052	0.244 ± 0.113

With compound FR5*	0.192 ± 0.007	0.228 ± 0.009	0.245 ± 0.004	0.177 ± 0.005	0.165 ± 0.002	0.204 ± 0.003	0.147 ± 0.003
Without compound	0.584 ± 0.002	0.355 ± 0.038	0.244 ± 0.006	0.254 ± 0.003	0.391 ± 0.003	0.309 ± 0.114	0.202 ± 0.099

With compound FR13	0.255 ± 0.003	0.206 ± 0.004	0.257 ± 0.005	0.215 ± 0.004	0.182 ± 0.002	0.245 ± 0.003	0.155 ± 0.004
Without compound	0.304 ± 0.003	0.284 ± 0.006	0.355 ± 0.003	0.254 ± 0.003	0.277 ± 0.001	0.539 ± 0.052	0.211 ± 0.002

With 2(5H)-furanone (+ve control)	0.120 ± 0.004	0.092 ± 0.004	0.106 ± 0.004	0.116 ± 0.004	0.119 ± 0.002	0.124 ± 0.003	0.105 ± 0.004
Without compound	0.262 ± 0.003	0.487 ± 0.003	0.486 ± 0.004	0.559 ± 0.015	0.564 ± 0.002	0.377 ± 0.122	0.216 ± 0.005

With BHI broth (−ve control)	0.057 ± 0.038	0.050 ± 0.006	0.021 ± 0.002	0.069 ± 0.020	0.095 ± 0.012	0.084 ± 0.006	0.084 ± 0.006
Without compound	0.276 ± 0.004	0.369 ± 0.056	0.257 ± 0.024	0.363 ± 0.079	0.316 ± 0.056	0.304 ± 0.003	0.163 ± 0.001

OD >0.24 is positive biofilm former isolate.

OD >0.12–<0.24 is weak biofilm former isolate.

OD <0.12 is negative biofilm former isolate.

*represents the most active compound.

**Table 2:** Antibiofilm activity of potential compounds of 139SI against Gram-positive clinical isolates.

Experimental treatment	*Staphylococcus aureus *	*Streptococcus agalactiae *	Group G Streptococci	*Streptococcus pyogenes *	*Streptococcus pneumoniae *	Biofilm-forming strain *S. aureus * (ATCC 25923)	Nonbiofilm-forming strain *E. coli * (ATCC 25922)
	OD ± SD	OD ± SD	OD ± SD	OD ± SD	OD ± SD	OD ± SD	OD ± SD
With compound FR4	0.254 ± 0.004	0.205 ± 0.005	0.199 ± 0.010	0.191 ± 0.003	0.230 ± 0.004	0.246 ± 0.004	0.186 ± 0.004
Without compound	0.484 ± 0.008	0.215 ± 0.002	0.253 ± 0.002	0.304 ± 0.003	0.371 ± 0.002	0.395 ± 0.003	0.216 ± 0.073

With compound FR5*	0.224 ± 0.004	0.166 ± 0.005	0.158 ± 0.002	0.153 ± 0.005	0.224 ± 0.004	0.166 ± 0.005	0.158 ± 0.002
Without compound	0.368 ± 0.028	0.304 ± 0.003	0.377 ± 0.122	0.363 ± 0.079	0.445 ± 0.042	0.030 ± 0.038	0.224 ± 0.005

With compound FR13	0.208 ± 0.004	0.205 ± 0.004	0.195 ± 0.004	0.216 ± 0.004	0.235 ± 0.004	0.254 ± 0.004	0.165 ± 0.004
Without compound	0.404 ± 0.003	0.276 ± 0.056	0.262 ± 0.003	0.257 ± 0.024	0.277 ± 0.002	0.378 ± 0.003	0.254 ± 0.003

With 2(5H)-furanone (+ve control)	0.119 ± 0.002	0.123 ± 0.003	0.116 ± 0.004	0.117 ± 0.001	0.121 ± 0.001	0.107 ± 0.004	0.116 ± 0.002
Without compound	0.257 ± 0.003	0.216 ± 0.005	0.206 ± 0.055	0.202 ± 0.099	0.214 ± 0.030	0.216 ± 0.005	0.211 ± 0.002

With BHI broth (−ve control)	0.057 ± 0.038	0.050 ± 0.006	0.021 ± 0.002	0.069 ± 0.020	0.095 ± 0.012	0.084 ± 0.006	0.084 ± 0.006
Without compound	0.254 ± 0.003	0.243 ± 0.045	0.211 ± 0.002	0.243 ± 0.045	0.270 ± 0.042	0.243 ± 0.002	0.208 ± 0.074

OD >0.24 is positive biofilm former isolate.

OD >0.12–<0.24 is weak biofilm former isolate.

OD <0.12 is negative biofilm former isolate.

*The most active compound.

**Table 3:** Liver and renal function tests among male rats.

	Male experiment groups			Control (reference range)	International unit (IU)
	Vehicle (0.9% NaCl)	Low dose (2 gm/Kg)	High dose (4 gm/Kg)		
Liver function test					
Total protein	57.50 ± 2.45*	67.33 ± 1.72	65.83 ± 2.79	64–82	g/L
Albumin	10.00 ± 2.04*	12.46 ± 1.35*	16.31 ± 5.98*	35–50	g/L
Globulin	42.33 ± 4.77*	54.33 ± 1.83*	50.83 ± 4.14*	23–35	g/L
Total bilirubin	3.66 ± 0.76	0.31 ± 0.47	3.75 ± 1.12	3–17	*µ*mol/L
Conjugate bilirubin	1.50 ± 0.34	1.33 ± 0.21	1.50 ± 0.34	0–3	*µ*mol/L
Alkaline phosphatase	256.16 ± 29.30*	229.66 ± 22.48*	195.50 ± 22.10*	50–136	IU/L
Alanine aminotransferase	74.50 ± 10.59*	83.16 ± 5.67*	107.16 ± 16.66*	30–65	IU/L
Aspartate aminotransferase	210.50 ± 22.81*	188.16 ± 20.13*	201.16 ± 17.64*	15–37	IU/L
G-Glutamyltransferase	11.00 ± 3.35*	8.33 ± 2.06*	10.33 ± 4.27*	15–85	IU/L

Renal function test					
Sodium	139.00 ± 1.12	138.66 ± 0.91	137.83 ± 1.40	136–145	mmol/L
Potassium	6.93 ± 0.79*	6.03 ± 0.25*	6.45 ± 0.72*	3.6–5.2	mmol/L
Chloride	101.83 ± 1.01	102.00 ± 1.03	102.16 ± 1.24	100–108	mmol/L
Carbon dioxide	28.05 ± 1.50	29.21 ± 0.72	26.41 ± 1.38	21.0–30.0	mmol/L
Anion gap	16.16 ± 1.37	14.76 ± 0.74	16.76 ± 2.11	10–20	mmol/L
Urea	7.78 ± 0.81*	8.71 ± 0.71*	9.35 ± 1.77*	2.5–6.4	mmol/L
Creatinine	18.16 ± 2.38*	21.00 ± 3.29*	20.00 ± 4.67*	61.9–115	*µ*mol/L

Values are expressed as the standard error mean ± S.E.M. and the significant value was at *P* < 0.05.

*Values that are above or below the control reference range.

g/L: gram per liter, *μ*mol/L: micromole per liter, and IU/L: international unit per liter.

**Table 4:** Liver and renal function tests among female rats.

	Female experiment groups			Contro (reference range)	International unit (IU)
	Vehicle (0.9% NaCl)	Low dose (2 gm/Kg)	High dose (4 gm/Kg)		
Liver function test					
Total protein	61.00 ± 3.81*	71.66 ± 0.95	63.16 ± 2.46*	64–82	g/L
Albumin	15.83 ± 3.00*	13.66 ± 1.02*	16.66 ± 5.74*	35–50	g/L
Globulin	45.83 ± 3.77*	57.50 ± 1.64*	48.16 ± 4.85*	23–35	g/L
Total bilirubin	6.83 ± 2.15	2.83 ± 0.83*	4.66 ± 1.76	3–17	*µ*mol/L
Conjugate bilirubin	1.83 ± 0.40	1.33 ± 0.33	1.50 ± 0.34	0–3	*µ*mol/L
Alkaline phosphatase	156.33 ± 38.10*	136.50 ± 21.53*	140.00 ± 23.42*	50–136	IU/L
Alanine aminotransferase	67.50 ± 11.44*	67.50 ± 3.19*	82.33 ± 16.32*	30–65	IU/L
Aspartate aminotransferase	150.33 ± 19.10*	196.66 ± 19.69*	196.83 ± 24.25*	15–37	IU/L
G-Glutamyltransferase	5.66 ± 0.71*	4.50 ± 0.56*	7.66 ± 1.68*	15–85	IU/L

Renal function test					
Sodium	139.50 ± 0.99	138.00 ± 1.06	138.50 ± 0.84	136–145	mmol/L
Potassium	6.80 ± 0.59*	6.10 ± 0.65*	5.56 ± 0.25*	3.6–5.2	mmol/L
Chloride	96.66 ± 4.70*	100.66 ± 1.05	98.50 ± 2.61*	100–108	mmol/L
Carbon dioxide	24.68 ± 1.04	27.03 ± 1.35	26.66 ± 1.37	21.0–30.0	mmol/L
Anion gap	24.68 ± 1.04*	27.03 ± 1.35*	26.66 ± 1.37*	10–20	mmol/L
Urea	7.50 ± 1.12*	7.15 ± 0.32*	7.23 ± 0.64*	2.5–6.4	mmol/L
Creatinine	23.33 ± 2.76*	24.50 ± 3.78*	28.66 ± 1.20*	61.9–115	*µ*mol/L

Values are expressed as the standard error mean ± S.E.M. and the significant value was at *P* < 0.05.

*Values that are above or below the control reference range.

g/L: gram per liter, *μ*mol/L: micromole per liter, and IU/L: international unit per liter.

**Table 5:** Complete blood count and differential blood count tests among male rats.

	Male experimental groups			Control (reference range)	International unit (IU)
	Vehicle (0.9% NaCl)	Low dose (2 gm/Kg)	High dose (4 gm/Kg)		
Complete blood count (CBC) test					
Hemoglobin (HGB)	144.333 ± 3.323	147.166 ± 3.709	139.500 ± 4.295	130–170	g/L
Hematocrit (HCT)	0.421 ± 0.006	0.465 ± 0.013	0.466 ± 0.016	0.40–0.50	L/L
Red blood cells (RBC)	5.583 ± 0.241*	5.733 ± 0.187*	6.173 ± 0.246*	4.50–5.50	10^12^/L
Mean corpuscular volume (MCV)	57.500 ± 1.543*	65.666 ± 2.011*	65.833 ± 3.590*	77–97	fL
Mean corpuscular hemoglobin (MCH)	23.050 ± 0.755*	23.333 ± 1.227*	25.083 ± 1.549*	27.0–32.0	Pg
Mean corpuscular hemoglobin concentration (MCHC)	326.000 ± 1.807	328.500 ± 1.522	337.666 ± 2.245	315–345	g/L
Red blood cell distribution width (RDW)	12.483 ± 0.335	12.600 ± 0.265	13.683 ± 0.514	11.6–14.0	%
White blood cells (WBC)	5.516 ± 0.286	6.216 ± 0.613	11.333 ± 0.792*	4.0–10.0	10^9^/L
Platelet	247.833 ± 12.605	297.333 ± 20.397	411.333 ± 19.022*	150–400	10^9^/L

Differential blood count test					
Neutrophil	5.666 ± 0.714	8.500 ± 0.763*	9.166 ± 1.077*	2.00–7.00	10^9^/L
Lymphocyte	2.833 ± 0.307	3.666 ± 0.421*	7.166 ± 0.477*	1.00–3.00	10^9^/L
Monocyte	1.183 ± 0.079*	1.650 ± 0.168*	2.366 ± 0.164*	0.20–1.00	10^9^/L
Eosinophil	0.188 ± 0.032	0.270 ± 0.024	0.258 ± 0.054	0.02–0.50	10^9^/L
Basophil	0.011 ± 0.007*	0.026 ± 0.010	0.045 ± 0.010	0.02–0.10	10^9^/L

Values are expressed as the standard error mean ± S.E.M. and the significant value was at *P* < 0.05.

*Values that are above or below the control reference range.

g/L: gram per liter, L/L: liter per liter, fL: femtoliters, pg: pictogram, and %: percentage.

**Table 6:** Complete blood count and differential blood count tests among female rats.

	Female experimental groups			Control (reference range)	International unit (IU)
	Vehicle (0.9% NaCl)	Low dose (2 gm/Kg)	High dose (4 gm/Kg)		
Complete blood count (CBC) test					
Hemoglobin (HGB)	140.666 ± 2.577	135.000 ± 2.840	150.833 ± 4.158	130–170	g/L
Hematocrit (HCT)	0.433 ± 0.009	0.458 ± 0.015	0.455 ± 0.011	0.40–0.50	L/L
Red blood cells (RBC)	5.566 ± 0.252*	6.350 ± 0.232*	7.300 ± 0.343*	4.50–5.50	10^12^/L
Mean corpuscular volume (MCV)	63.500 ± 2.667	60.333 ± 2.564	60.500 ± 2.753	77–97	fL
Mean corpuscular hemoglobin (MCH)	21.216 ± 0.820*	23.683 ± 0.603*	20.500 ± 0.940*	27.0–32.0	pg
Mean corpuscular hemoglobin concentration (MCHC)	328.833 ± 2.329	335.833 ± 2.358	343.666 ± 5.129	315–345	g/L
Red blood cell distribution width (RDW)	12.533 ± 0.289	12.500 ± 0.163	12.866 ± 0.401	11.6–14.0	%
White blood cells (WBC)	5.983 ± 0.411	8.216 ± 0.503	10.916 ± 0.925*	4.0–10.0	10^9^/L
Platelet	322.833 ± 28.703	375.833 ± 17.284	433.666 ± 27.690*	150–400	10^9^/L

Differential blood count test					
Neutrophil	6.000 ± 0.966	10.166 ± 0.703*	11.166 ± 0.600*	2.00–7.00	10^9^/L
Lymphocyte	3.333 ± 0.421*	2.833 ± 0.477	6.500 ± 1.056*	1.00–3.00	10^9^/L
Monocyte	1.350 ± 0.133*	1.800 ± 0.146*	2.016 ± 0.280*	0.20–1.00	10^9^/L
Eosinophil	0.333 ± 0.030	0.246 ± 0.049	0.316 ± 0.030	0.02–0.50	10^9^/L
Basophil	0.015 ± 0.007*	0.030 ± 0.010	0.040 ± 0.013	0.02–0.10	10^9^/L

Values are expressed as the standard error mean ± S.E.M. and the significant value was at *P* < 0.05.

*Values that are above or below the control reference range.

g/L: gram per liter, L/L: liter per liter, fL: femtoliters, pg: pictogram, and %: percentage.
